# "Janus" Cyclic Peptides: A New Approach to Amyloid Fibril Inhibition?

**DOI:** 10.1371/journal.pone.0057437

**Published:** 2013-02-20

**Authors:** Nevena Todorova, Levi Yeung, Andrew Hung, Irene Yarovsky

**Affiliations:** 1 School of Aerospace, Mechanical and Manufacturing Engineering, RMIT University, Melbourne, Victoria, Australia; 2 School of Applied Science, RMIT University, Melbourne, Victoria, Australia; 3 Health Innovations Research Institute, RMIT University, Melbourne, Victoria, Australia; University of Akron, United States of America

## Abstract

Cyclic peptides are increasingly being shown as powerful inhibitors of fibril formation, and have the potential to be therapeutic agents for combating many debilitating amyloid-related diseases. One such example is a cyclic peptide derivative from the human apolipoprotein C-II, which has the ability to inhibit fibril formation by the fibrillogenic peptide apoC-II(60–70). Using classical molecular dynamics and electronic structure calculations, we were able to provide insight into the interaction between the amyloidogenic peptide apoC-II(60–70) and its cyclic derivative, cyc(60–70). Our results showed that cyc(60–70) induced increased flexibility in apoC-II(60–70), suggesting that one mechanism by which cyc(60–70) inhibits fibrillisation is by destabilising apoC-II(60–70) structure, rendering it incapable of adopting fibril favouring conformations. In contrast, cyc(60–70) shows less flexibility upon binding to apoC-II(60–70), which is predominantly mediated by hydrophobic interactions between the aromatic rings of the peptides. This effectively creates a cap around the fibril-forming region of apoC-II(60–70) and generates an outer hydrophilic shell that discourages further apoC-II(60–70) peptide self-association. We showed that apoC-II(60–70) exhibited stronger binding affinity for the hydrophobic face of cyc(60–70) and weakest binding affinity for the hydrophilic side. This suggests that cyc(60–70) can be an effective fibril inhibitor due to its amphipathic character, like that of the "Janus"-type particles. This property can be exploited in the design of specific inhibitors of amyloid fibril formation.

## Introduction

Insoluble protein aggregates are the key feature of amyloid deposits responsible for a range of debilitating conditions, such as Alzheimer's disease, Parkinson's disease and type-II diabetes. The oligomeric intermediates and pre-formed fibrils have been shown to be the toxic species in the disease progression 1]. The development of peptide agents that inhibit or reverse the misfolding and aggregation of proteins by targeting the protein-protein interfaces inherent in amyloid fibrils is a useful approach to combat these crippling diseases. Specifically, cyclic peptides (CPs) have been shown to be good peptide inhibitors of amyloid formation 2,3,4] and have the ability to reduce pre-fibrilar toxicity 5]. Many hormones, antibiotics and toxins such as cyclosporine, bacitracin and α-amanitin, exist naturally as CPs 6]. CPs are metabolised at a slower rate due to their resistance towards chemical degradation. However, they are excreted more rapidly than their linear counterparts as a result of their hydrophobic affinity. Several peptide cyclisation strategies have been established that enable the development of cyclic peptides through disulfide bonds and lactam bridges. The cyclisation of Aβ(1–28) at residues 17 and 21 via a lactam bridge has been shown to inhibit fibril formation by Aβ(1–40) and reduce its cytotoxicity 3]. In another study, macrocycles containing the pentapeptide VQIVY were found to suppress the onset of aggregation of tau-derived peptides, AcPHF6 4]. It was proposed that a pair of macrocycles cap the peptide interfaces responsible for aggregation and block the growth of β-sheets.

We have recently shown that a cyclic peptide derivative of human apolipoprotein C-II (apoC-II) can be an effective inhibitor of fibril formation by its linear counterpart, apoC-II(60–70) 2]. ApoC-II is a 79 residue protein member of the very low density lipoproteins and a physiological activator of lipoprotein lipase. In lipid-depleted environment apoC-II self-assembles into fibrils with all of the defining characteristics of amyloid fibrils 7,8]. Amyloid fibrils formed by apoC-II initiate early events in heart disease, including the induction of the macrophage inflammatory response. It was also shown that residues 60 to 70 of apoC-II, make apoC-II(60–70) peptide, which retains its ability to form fibrils 9]. Our previous molecular dynamics (MD) simulations of apoC-II(60–70) in solution showed structural tendencies towards the formation of β-hairpin-like conformations, in which the N- and C-termini are generally positioned in close proximity 10,11]. These structures may be initiating the earliest intermolecular interactions on the aggregation pathway in the fibril forming process. Recently, we showed that cyc(60–70), a cyclised form of apoC-II(60–70), formed by disulphide cross-linking of cysteine residues added at each end of the peptide, inhibited fibril formation by apoC-II(60–70) and apoC-II(56–76) 2]. NMR spectroscopy revealed a well-defined cyc(60–70) structure exhibiting a hydrophilic face and a more hydrophobic face containing the Met60, Tyr63, Ile66 and Phe67 side chains, while the MD simulations identified an inherently flexible central region. However, although the structure of cyc(60–70) has been well characterised and ThT experimental data indicates that cyc(60–70) disrupts fibril formation of apoC(60–70), albeit its ineffectiveness for the full-length protein, the cyclic peptide serves as an ideal prototype for the development of possible inhibiting peptide agents which requires its mechanisms of inhibition to be properly identified.

In this article we used classical molecular dynamics simulations of apoC-II(60–70) peptide in the presence of cyc(60–70) to investigate the structure, dynamics and interactions between the two peptides. As a first step towards understanding atomic-level interactions between cyclic and linear peptides we focused on the simplest possible system, which enables us to examine some of the mechanisms responsible for the cyclic peptide's inhibitory efficacy. The free energy of dissociation and interaction enthalpies were determined using potential of mean force and quantum mechanical calculations to identify the favourable sites and the mechanism of binding of apoC-II(60-70) to cyc(60–70).

## Results/Discussion

### ApoC-II(60–70) - cyc(60–70) heterodimer: structure and dynamics

Using molecular dynamics simulations 2.8 µs of conformational statistics was collected for data analysis. First, cluster analysis of the ensemble trajectory (frames taken at 240 ps intervals) was performed using the single linkage method, where a structure was added to a cluster when its RMSD to any element of the cluster was less than a selected cut-off value. A cut-off value was chosen which resulted in elucidation of <10 clusters and/or resulted in the largest cluster containing no more than 30% of the conformational population. This was reached at a cut-off of 2 Å (backbone only).

**Figure 1:**
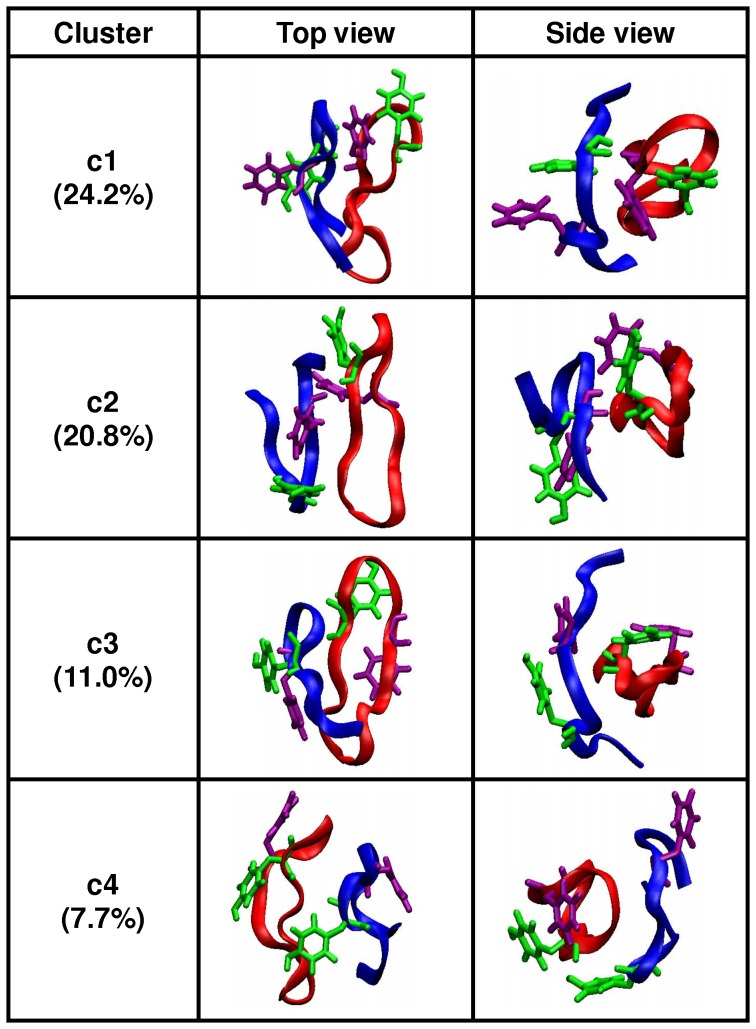
Most populated structures determined by clustering analysis. Ribbon and explicit aromatic ring representations of the four most populated clusters (green – Tyr63; purple – Phe67). The apoC-II(60–70) peptide is shown in blue and cyc(60–70) is shown in red.

The clustering analysis over the heterodimer complex identified 584 clusters. The four most populated clusters had 24.2% (c1), 20.8% (c2), 11.0% (c3) and 7.7% (c4), structures dispersed throughout the ensemble trajectory (data not shown). The representative structures of the four most populated clusters are shown in Figure 1. To aid clarity for discussion, the apoC-II(60–70) peptide of each cluster is referred to as lin-c1, lin-c2, lin-c3 and lin-c4, respectively.

**Figure 2:**
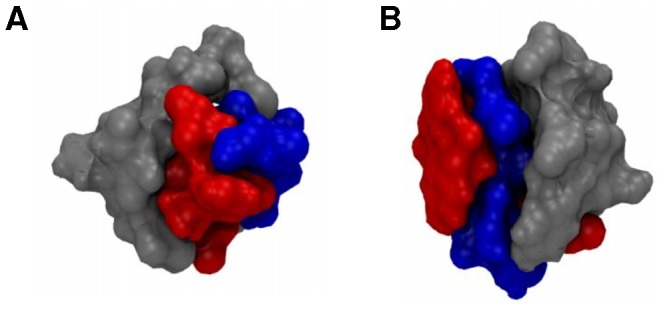
ApoC-II(60-70) binding preference sites on cyc(60-70). Surface representations showing the binding preference sites for structures: (**A**) c1 and (**B**) c4. Cyc(60–70) hydrophobic face is shown in red and the hydrophilic face in blue, while the apoC-II(60–70) peptide is coloured grey.

The representative apoC-II(60–70) peptide structures in clusters c1 and c3 (lin-c1 and lin-c3) exhibited extended coil conformations with a noticeable separation between the N- and C- termini. The peptides adopted significantly different conformations compared to the β-hairpin-like structures observed in monomeric and mutated apoC-II(60–70) in solution 10,11]. In particular, a broader β-turn was adopted to adjust to the significant increase of the distance between the N- and C- termini. This enabled a larger contact area between apoC-II(60–70) peptide and cyc(60–70). Lin-c1 preferentially docked to the hydrophobic face of cyc(60–70), as illustrated in Figure 2A. The four aromatic side-chains (Tyr63 and Phe67) of the two peptides formed a distinct hydrophobic cluster, while the hydrophilic residues remained exposed to the solvent. In contrast, lin-c2 and lin-c4 exhibited β-hairpin-like structures, suggesting that cyc(60–70) can induce conformational changes in apoC-II(60–70). Lin-c4 was observed to dock to the hydrophilic face of cyc(60–70) (Figure 2B) however this arrangement was relatively short lived, as can be seen from the low population of this cluster.

**Figure 3:**
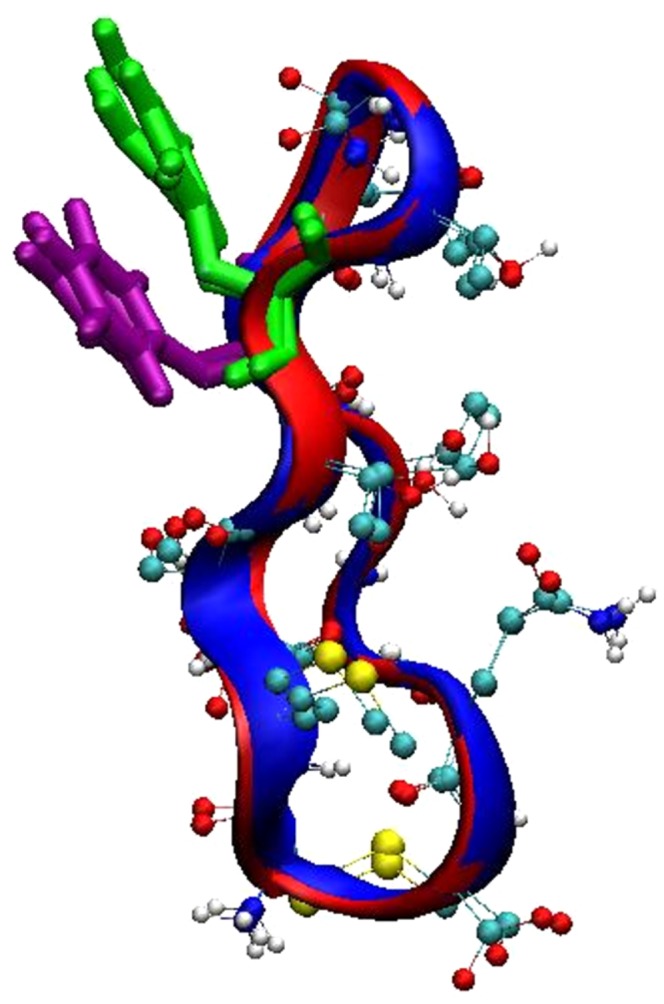
Cyc(60–70) structure overlay of monomeric and heterodimeric state. Superimposition of the cyc(60–70) structures taken from the most populated cluster, c1 (red) and monomeric cyc(60–70) 2] (blue). The aromatic residues are also shown; Tyr63 in green and Phe67 in purple.

The β-turn region in the cyclic peptide consisting of residues Thr64, Gly65 and Ile66 was found to be similar to that observed in our previous study of the monomeric cyc(60–70) 2]. In the heterodimer complex, cyc(60–70) peptide retained its elongated conformation, stabilised by the π-stacking between the aromatic rings. The cyc(60–70) peptide was previously characterised as a flexible molecule, exhibiting a dynamic hydrophobic core with a cluster population size of 13.8% 2]. However in the linear-cyclic heterodimer complex, the cluster analysis of cyc(60–70) alone, found the most stable cluster with 66.1% population (1.3 Å cut-off). This suggests that dimerisation induced structural stability to cyc(60–70). In all four most populated dimer clusters, cyc(60–70) has a structure similar to the monomeric cyc(60–70) in solution with a backbone RMSD value between the dimerised and free peptide no higher than 0.91 Å (Figure 3).

The cyclic peptide was previously shown to have an average of 4.7 internal H-bonds 2]. However in a dimer form, the cyclic peptide was found to be significantly more stable, accompanied by an increase in intramolecular hydrogen bonding to 5.7 H-bonds per molecule. The H-bond statistics of the cyclic peptide (in the dimer form) is shown in Figure S2 of Supporting Information.

**Figure 4:**
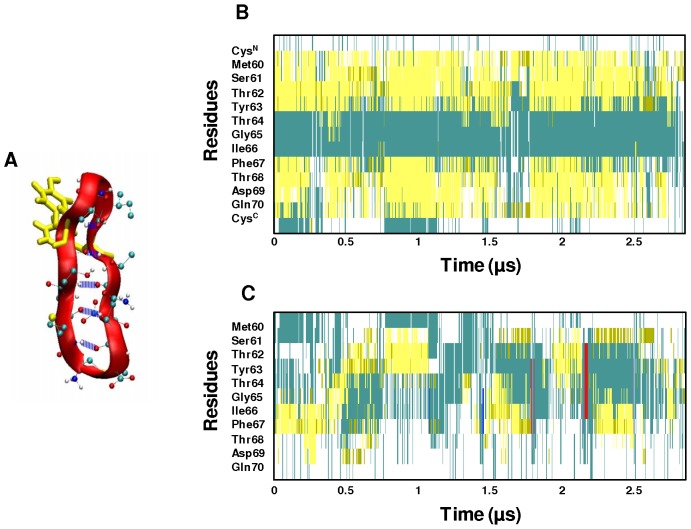
Structural evolution of cyc(60–70) and apoC-II(60–70). (**A**) Ribbon and CPK representation of cyc(60–70) showing the persistent intra-molecular backbone hydrogen bonds in blue. The secondary structure evolution of (**B**) cyc(60–70) and (**C**) apoC-II(60–70) over 2.8 µs of simulation. The secondary structure colour codes: cyan – turn; yellow – extended conformation; green – hydrogen bridge; white – coil; blue – 3-10 helix; red – π-helix.

Inspection of Figure S1 reveals the presence of persistent intramolecular H-bonds in cyc(60–70) formed between Gln70(NH)-Met60(O), Thr68(NH)-Thr62(O), Ile66(NH)-Thr64(O), Thr64(NH)-Ile66(O), Thr62(NH)-Thr68(O), Ser61(OG,HG)-Asp69(OD1), Ser61(OG,HG)-Asp69(OD2) and Met60(NH)-Gln70(O). It is important to note that six of the eight identified persistent H-bonds occur between the backbone of the two-strands (Figure 4A), and contribute to the structural stability of the cyc(60–70) structure.

The secondary structure of the peptides was analysed by VMD using the STRIDE algorithm, which utilises hydrogen bond energy and mainchain dihedral angles in addition to hydrogen bond distances to classify the structure 12]. The secondary structure evolution of the cyc(60–70) (Figure 4B) indicates that the peptide retains its characteristic strand-turn-strand motif throughout the 2.8 µs of simulation. The cyclic peptide was found to undergo minor structural changes in the two strands, Met60-Tyr63 and Phe67-Gln70, howbeit predominantly adopted extended conformations. Our study of monomeric cyc(60–70) identified a flexible central core region 2], however, in a heterodimeric state, the central core region of the cyclic peptide is less flexible. This confirms that the bound apoC-II(60–70) has a stabilising effect on the cyc(60–70) structure.

In contrast, inspection of Figure 4C reveals that apoC-II(60–70) peptide exhibits a high degree of conformational changes when bound to cyc(60–70). Interestingly, apoC-II(60–70) was previously shown to be highly stable in the isolated monomeric state 10]. However in a dimer complex, apoC-II(60–70) was observed to be much more dynamic, adopting a wide range of conformations. *This indicates that whilst the presence of apoC-II(60*–*70) reduces the flexibility of cyc(60*–*70), the cyc(60*–*70) peptide induces increased flexibility in apoC-II(60*–*70)*. It can be hypothesised that cyclic induced structural lability of apoC-II(60–70) prevents it from adopting stable conformations which favour amyloid aggregate initiation. This hypothesis is in line with our findings on oxidised apoC-II(60–70), where increased structural flexibility and dynamics were the key factors preventing this peptide to form fibrils 10].

To further characterise the flexibility of apoC-II(60–70), the H-bond existence profile was calculated for the duration of the trajectory and shown in Figure S2 of Supporting Information. The results indicated the lack of persistent H-bonds except for the short simulation periods of less than 0.3 µs (1.5 µs to 1.8 µs and 2.2 µs to 2.5 µs) of the c2 apoC-II(60–70) structure (Figure 1, lin-c2). The apoC-II(60–70) peptide in this cluster adopts a β-hairpin conformation stabilised by intramolecular hydrogen bonding similar to that observed in our MD studies of isolated apoC-II(60–70) in solution 10,11]. The following persistent H-bonds were identified: Asp69(NH)-Ser61(OG), Thr68(NH)-Met60(O), Thr68(NH)-Ser61(OG), Ile66(NH)-Thr62(OG1), Gly65(NH)-Thr62(O), Thr62(NH)-Ile66(O), Ser61(OG,HG)-Asp69(OD2) and Ser61(OG,HG)-Asp69(O), where Thr68(NH)-Met60(O), Gly65(NH)-Thr62(O) and Thr62(NH)-Ile66(O) are H-bonds between the peptide's backbone, indicating structural stability. This is a significant observation because the lack of these backbone H-bonds enables the linear peptide to adopt a wider range of conformations, including those observed in lin-c1 and lin-c3 shown in Figure 1. Furthermore, the apoC-II(60–70) peptide in a heterodimer complex with cyc(60–70) on average possesses 2.8 H-bonds per analysed frame, which is significantly lower than the average of 4.2 H-bonds per frame identified for isolated apoC-II(60–70) in solution 10].

**Figure 5:**
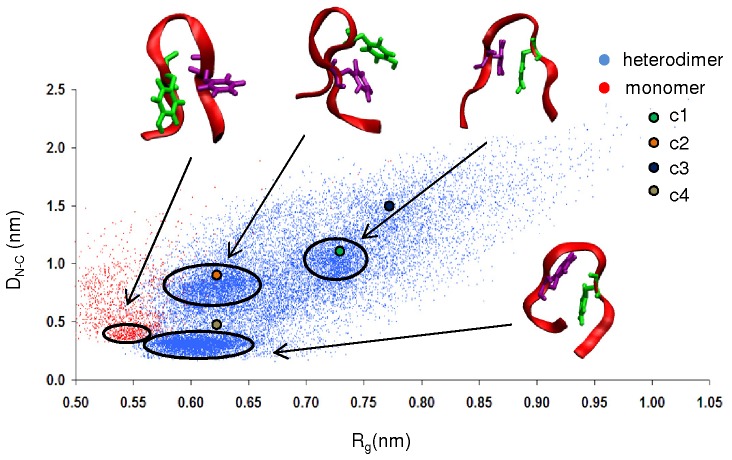
ApoC-II(60–70) structural characteristics in free and cyc(60–70) bound states. End-to-end distance with respect to the radius of gyration of apoC-II(60–70) peptide in its monomeric state (red data points 10]) and as a heterodimer bound to cyc(60–70) (blue data points). Ribbon representations of the peptides corresponding to the indicated regions of sampled conformational space are shown as insets. The positions of the cluster representative structures are illustrated with coloured circles: c1 (green), c2 (orange), c3 (dark blue) and c4 (tan).

To further characterise the overall structure of apoC-II(60–70) peptide in the dimer complex, the distance between the N- and C- termini, *D_N-C_* (peptide extension) and its radius of gyration, *R_g_* (sphericity) were calculated. A plot of the peptide extension, *D_N-C_* with respect to *R_g_* shown in Figure 5 reveals clusters of data points centering around four (*D_N-C_*, *R_g_*) regions. The cluster shown in red indicates that apoC-II(60–70) in its monomeric state frequently samples the conformational basin around *D_N-C_*  = 0.45 and *R_g_* = 0.55 nm (identified as (0.45, 0.55) for discussion). In this basin, the peptide adopts a β-hairpin with the aromatic side-chains facing one side of the peptide, forming a hydrophobic region.

In line with our previous observations the presence of cyc(60–70) renders apoC-II(60107270) more dynamic, whereby it frequently samples three conformational basins around (0.30, 0.60), (0.75, 0.63) and (1.00, 0.73) regions, with significant structural variations evident by the spread of the data points. This confirms that the peptide is able to adopt a wide range of conformations, as seen by the cluster analysis in Figure 1. The positions of the cluster representative structures are also presented in Figure 5. The results show that the most frequently sampled structures are within the outlined regions or very close by. This affirms that the cut-off used for the clustering analysis is appropriate to identify conformations with similar structural features.

### The heterodimer exhibits propensity to form hydrophobic clusters

Hydrophobic interactions, especially those between aromatic side-chains, have been shown to play an important role in the formation 13,14] and stability 15,16] of amyloid fibrils. We have established that two aromatic rings (Tyr63 and Phe67) are crucial for the aggregation of apoC-II(60–70) and there are distinct differences in the aromatic side-chain orientations between fibril-forming and fibril-inhibiting states of the peptide 10,11].

In our previous study we identified that under fibril-inhibiting conditions, the aromatic rings adopt conformations where Tyr63 and Phe67 lie on the same side of apoC-II(60–70) peptide, whereas structures obtained under fibril-favouring conditions have aromatic rings on opposing sides 10]. Monomeric cyc(60–70) was found to be very flexible, able to adopt both fibril-inhibiting and fibril-favouring aromatic rings arrangements 10]. The relative orientations of Tyr63 and Phe67 in apoC-II(60–70) and cyc(60–70) in the dimer complex was determined by calculating the average angle between the Cα-Cγ vector of Tyr63 and the Cα-Cγ vector of Phe67.

**Figure 6:**
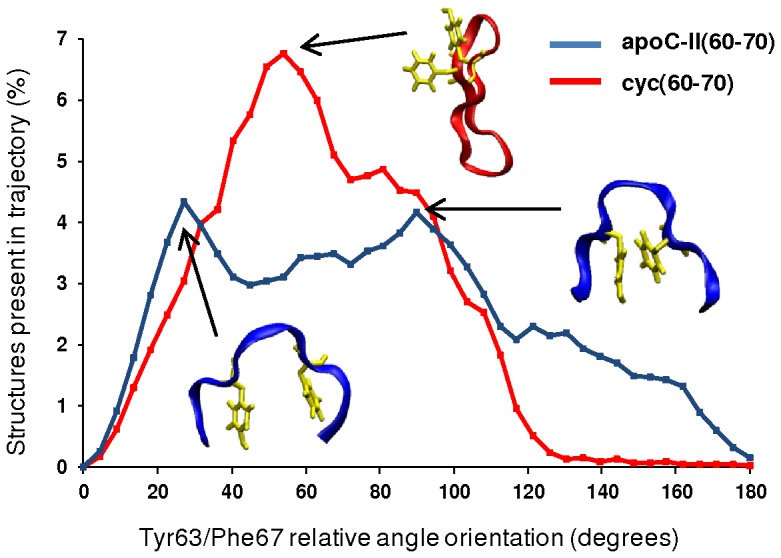
Aromatic ring orientation in apoC-II(60-70) and cyc(60 –**70).** A histogram of the Tyr63 and Phe67 relative aromatic ring orientation (X-axis) obtained from the 2.8 µs ensemble trajectory. Typical structures illustrating the relative ring orientations of cyc(60–70) (red) and apoC-II(60–70) (blue) are represented as insets.

Figure 6 shows the percentage of structures with respect to the relative aromatic ring orientation. A total of forty-one bins were used over the range 0–180°, with each data point corresponding to an angle interval of 4.5°. Angle<90° indicates both rings are on one side of the peptide, while angle >90° indicates that the rings are on the opposite sides. The results show that in the heterodimer, cyc(60–70) prefers a relative aromatic ring orientation of less than 90° (peak at 54°), indicating that it now exhibits stronger fibril-inhibiting characteristics. Moreover, the relative ring orientation in the apoC-II(60–70) part of the heterodimer also exhibits fibril-inhibiting characteristics. Two peaks were observed for apoC-II(60–70) at 27° and 90° with both structures similar to those shown in Figure 1 and 5, where fibril-inhibiting characteristics such as the widening of the β-hairpin and the increase in distance between the N- and C- termini are featured. The total percentage of structures with ring orientations within 0° to 90° for cyc(60–70) and apoC-II(60–70) was calculated to be 83% and 62%, respectively. This suggests that while being in proximity of each other both apoC-II(60–70) and cyc(60–70) favour the formation of a hydrophobic cluster, which prevents the hydrophobic regions to be exposed to interaction with other like-peptides and initiate aggregation.

### Cyc(60–70) – apoC-II(60–70) binding energetics

**Figure 7:**
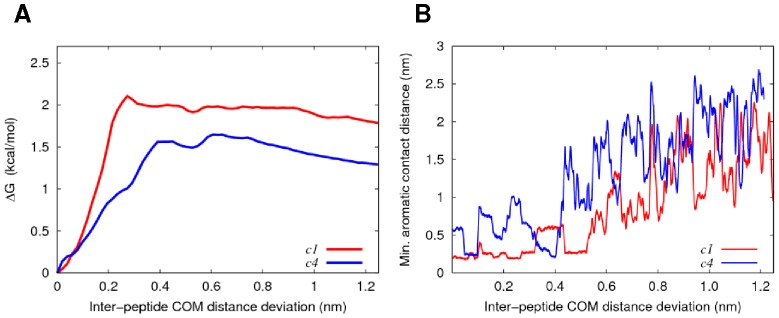
PMF and inter-peptide COM distance profiles from umbrella sampling simulations. (**A**) PMF profiles for the dissociation of apoC-II(60–70) peptide from the hydrophobic (red) and hydrophilic (blue) face of cyc(60–70). (**B**) Minimum contact distance between the aromatics as a function of centre-of-mass separation for apoC-II(60–70) and cyc(60–70). For clarity, the moving average for each profile is shown in red and blue, respectfully.

Potential of mean force (PMF) profiles were acquired to investigate the dissociation free energies of apoC-II(60–70) bound to the hydrophobic and hydrophilic face of cyc(60–70) in solution, shown in Figure 7A. The representative structures of c1 and c4 were taken as the starting structures for the umbrella sampling simulations because they exhibited the alternative arrangements (Figure 2).

The profiles indicate that higher free energy (∼0.5 kcal/mol) is required to fully separate apoC-II(60–70) from the hydrophobic face compared to the hydrophilic face of cyc(60–70). The favourable arrangement and close contacts between the aromatic residues of apoC-II(60–70) and the hydrophobic face of cyc(60–70) is a contributing factor to the stronger binding affinity, as can be seen in Figure 7B. Shortly after the contact between the aromatic rings is lost, the PMF profiles exhibit a plateauing free energy suggesting a complete dissociation of the peptides.

**Figure 8:**
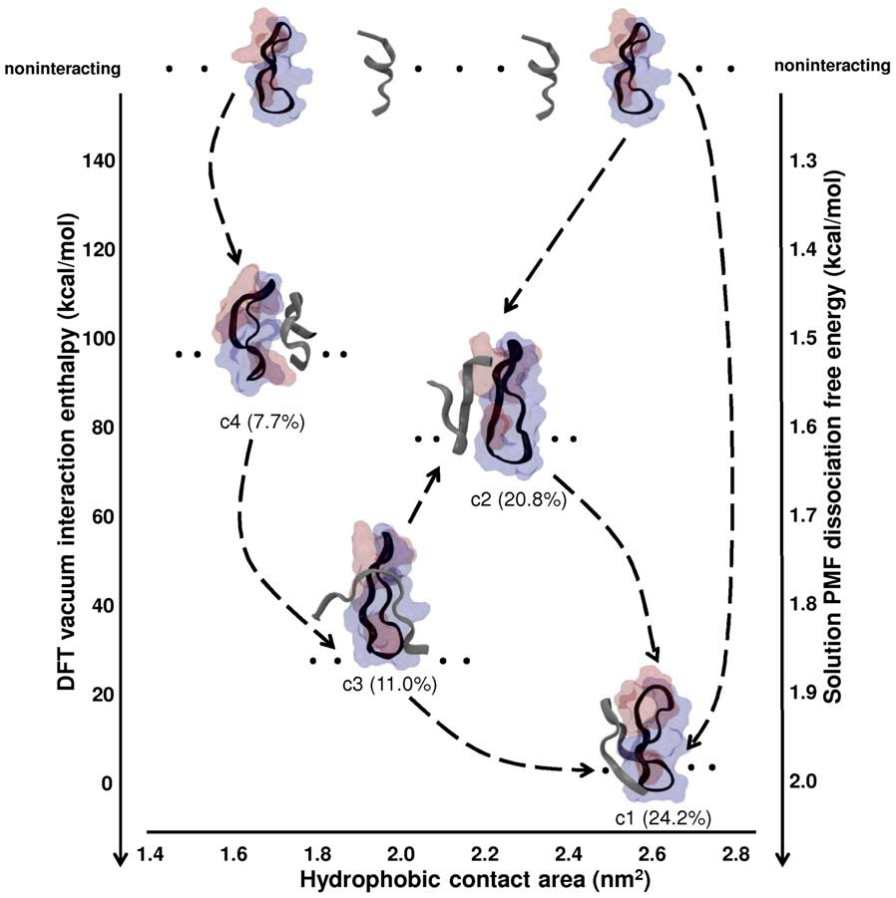
Conceptual association pathway for apoC-II(60–70) interacting with cyc(60–70). The four most populated clusters are shown with respect to their relative interaction enthalpies, PMF dissociation free energies (for clusters c1 and c4 only) and hydrophobic contact area. The respective cluster populations are also shown below each representative complex with dots indicating multiple possible structures. The connecting dashed lines demonstrate possible pathways, while the arrows point in the direction of stronger bound complexes. The hydrophobic residues of cyc(60–70) are represented as red surface, and hydrophilic residues in blue. The cyc(60–70) structure is shown with its hydrophobic face on the right and hydrophilic face on the left. The apoC-II(60–70) and cyc(60–70) conformation is shown in ribbon representation in grey and black, respectively.

In addition to the classical simulation-derived dissociation free energies discussed above, we have also used electronic structure calculations (DFT) to calculate the interaction enthalpies of the four most populated clusters of apoC-II(60–70) with cyc(60–70) in vacuo. This provides an independent (qualitative) measure of the relative dimerisation energies of the major conformational clusters. To account for the thermal disorder within the clusters, we calculated the interaction enthalpies of multiple structures (five per cluster, including the representative states). Figure 8 shows a conceptual illustration of possible association pathways for apoC-II(60–70) and cyc(60–70) with a diagram representing the conformational states, their relative populations and possible transitions with respect to the relative DFT interaction enthalpies to c1 (which has the lowest interaction enthalpy), PMF free energies and hydrophobic contact area. We find that the relative interaction enthalpies of the major clusters are consistent between classical (PMF) and DFT methods. This lends support to our treatment of c1 as the most stable dimer, as discussed in the following.

The dimeric conformation with the lowest enthalpy (c1) involves apoC-II(60–70) binding to the hydrophobic face of cyc(60–70), the former exhibiting a turn conformation with N- and C- termini well separated from each other. The large contact area between the peptides results in the lowest average interaction enthalpy. The interaction enthalpies of all other clusters are given relative to that of c1.

In addition to c1, other dimeric complexes are also formed during the simulation owing to the increased flexibility and dynamics of apoC-II(60–70) upon interaction with the cyclic peptide. Such interaction may contribute to a gain in configurational entropy in apoC-II(60–70) and lead to the formation of partial β-hairpin structures which results in less favourable binding with cyc(60–70) compared to c1. In particular, the lower hydrophobic contact areas observed for c2 and c3 produce weaker average interaction enthalpies, ∼35.4±10.1 to 91.7±8.8 kcal/mol higher than the most stable cluster c1. The degree of structural fluctuations within each cluster is reflected in the standard deviations of the calculated interaction enthalpy values, ∼13.3% and ∼8.2% for clusters c2 and c3, respectively. Other short-lived conformations, such as c4 (7.7% population, 97.6±13.6 kcal/mol relative interaction enthalpy to c1), involve contact via hydrophilic residues; here, the lack of hydrophobic contacts appears to play a role in its relatively low structural stability and also reflected in the high (∼22.5%) standard deviation of the calculated interaction enthalpies for this cluster. Thus, the higher flexibility, lower dimer interface area and weaker interaction energies of clusters c2, c3 and c4, suggest that they could be intermediate states ‘en route’ to the (more stable) c1 conformation.

Overall, cyc(60–70) induces structural flexibility in apoC-II(60–70) peptide, rendering it unable to self-associate. The cyclic peptide's elongated conformations help maximise hydrophobic interactions with apoC-II(60–70) that subsequently abolishes fibril formation by ‘capping’ the fibril seed assembly and thus generating a hydrophilic ‘shell’ that discourages fibril growth (Figure 2) similar to the capping effect of lipids shown previously 10,17,18,19].

## Conclusions

We have recently shown that a cyclic derivative is an effective inhibitor of fibril formation by apoC-II(60–70) peptide 2]. Understanding the mechanisms by which the cyclic molecules block the formation of amyloid fibrils can help design specific therapeutic agents to prevent amyloidosis.

In the current work, using classical molecular dynamics simulation and quantum calculations we were able to obtain insight in the interactions and binding affinity of the amyloidogenic apoC-II(60–70) peptide with its cyclic analogue cyc(60–70). Our results showed that cyc(60–70) induced increased flexibility in apoC-II(60–70), suggesting that in the presence of its cyclic analogue apoC-II(60–70) cannot adopt a stable fibril favouring conformation to initiate or further the fibril forming process. When bound to cyc(60–70), apoC-II(60–70) exhibited extended conformations dominated by turn structures, in contrast to the β-hairpin-like structures predominant in its monomeric form. We propose that the transient interactions of cyc(60–70) with the soluble fibrillogenic peptide reduces its ability to form fibrils by subtly shifting its dynamic equilibrium.

In turn, cyc(60–70) structure is stabilised upon binding to apoC-II(60–70) due to the entropically favourable formation of a large, contiguous hydrophobic region that maximises hydrophobic contacts between the fibrillogenic apoC-II(60–70) and cyc(60–70) peptide. Our association free energy and interaction enthalpy calculations showed that apoC-II(60–70) exhibits stronger binding affinity for the hydrophobic face of cyc(60–70) compared to the hydrophilic face. The preference of apoC-II(60–70) for association with the hydrophobic region of cyc(60–70), suggests that the cyclic peptide effectively protects the fibril-forming face of the amyloidogenic peptide, and exposes an outer hydrophilic region that discourages further peptide self-association.

To summarise, given the substoichiometric nature of cyc(60–70) activity 2], inhibition of fibril formation by cyc(60–70) can be postulated to occur via two processes: (i) cyc(60–70) induces structural lability in apoC-II(60–70) thus preventing it from adopting and maintaining fibril-forming conformations, and (ii) cyc(60–70) ‘blocks’ the hydrophobic region of the amyloidogenic peptide or its oligomeric aggregates thus inhibiting further growth of the fibril. The mechanism by which this amphipathic ‘two-faced’ Janus cyclic peptide inhibits fibril formation can be exploited for the design of new agents for prevention of amyloid fibril formation.

For future work, we will investigate the mechanisms of binding by cyc(60–70) to the preformed fibrillar aggregates of apoC-II(60–70), which were identified in our previous work as anti-parallel oligomers 11], as well as study the effects of cyc(60–70) on the full-length apoC-II protein.

## Methods

### Atomistic simulation of apoC-II(60–70) - cyc(60–70) heterodimer complex

The Gromacs 3.3 20] simulation package was employed for all MD simulations and the analysis of the results. The MD calculations were performed using the GROMOS96 force field and 43a1 parameter set under NPT conditions at 300 K. Two different starting structures of apoC-II(60–70) peptide (^60^MSTYTGIFTDQ^70^) were used to enhance the sampling of conformational space. A β-hairpin configuration of apoC-II(60–70) was taken from our previous study where it was identified as the most stable structure and a possible intermediate state to fibril formation 10]. The native conformation of the apoC-II(60–70) segment was extracted from the NMR structure of apoC-II (PDB code: 1SOH). The structure of the cyclic peptide, denoted cyc(60–70), was taken from our recent study where we identified its preferred conformation using NMR spectroscopy and MD simulations 2]. The peptide was cyclised through a disulphide bond by flanking the apoC-II(60–70) sequence with cysteine residues at each of the N- and C- termini. Four different starting cyclic-linear arrangements were modelled for each apoC-II(60–70) conformation to investigate the pathways of binding. Cyc(60–70) was placed ∼7 Å apart from the apoC-II(60–70) peptide to enable sufficient freedom for initial interactions. Schematics depicting the initial heterodimer arrangements are shown in Figure S3 of Supporting Information.

Each dimer was enclosed in a periodic box of 60 Å×60 Å×60 Å, solvated with SPC water molecules corresponding to a water density of ∼1.0 g/cm^3^. Systems were energy minimised using the steepest descent method to reduce steric clashes. Simulation conditions of constant temperature at 300 K and constant pressure of 1 bar was achieved by coupling the system to a Berendsen thermostat and barostat 21], respectively. The time-step for all simulations was set to 2 fs. Solvent equilibration simulations of 100 ps in length were performed on the energy minimised system where the peptide was restrained and the solvent was allowed to undergo free dynamics. Subsequently, un-restrained simulation of 350 ns for each system was performed and all simulation trajectories were then concatenated into an ensemble of 2.8 µs for data analysis.

### Potential of mean force calculations

We acquired potential of mean force (PMF) profiles to determine the free energy of dissociation (*ΔG*) for apoC-II(60–70) bound to the polar and non-polar faces of cyc(60–70). We employed umbrella sampling 22], as implemented in the Gromacs 3.3 simulation package, and weighted histogram analysis method (WHAM) 23] to determine the PMF, or ΔG, as a function of separation distance between the centres of mass of the two peptides. This method is applied to explicitly solvated systems and therefore accounts for the entropic contributions in the determination of the dissociation energies. In the present work, *ΔG* and PMF both refer to the free energy required to bring the two peptides from the dimeric, associated form to a separation *d* (nm). To obtain a PMF profile for each system, a series of simulations (windows) were performed in which the separations between the centre of mass (COM) of the peptides were restrained in each window by Hookean functions with force constants of 20 kcal/mol/Å^2^. Twenty-six windows were applied, with adjacent windows being separated by 0.5 Å. Each window was simulated for 20 ns. WHAM was subsequently applied to the accumulated umbrella sampling trajectories to remove the biasing potentials and obtain the unbiased PMF profiles.

### Interaction enthalpy calculations

Electronic structure calculations based on density functional theory (DFT) were used to calculate the peptide interaction enthalpies in vacuum of the most populated states sampled during the all-atom solvated simulations. To account for thermal effects peptide interaction enthalpies of multiple structures within each cluster were calculated (five per cluster, including the representative conformations) to obtain an ‘average’ interaction enthalpy for each highly populated state. The standard deviation of the energies directly reflects the degree of structural fluctuation within each cluster since the single point energy calculations were performed without geometry optimization to retain the atomic/structural arrangements of the system as obtained during the molecular dynamics simulations in solution.

The DFT code ONETEP 24] was employed, which combines linear scaling computational efficiency with accuracy that is comparable to traditional plane-wave DFT codes. Calculations conducted using ONETEP scale linearly with system size, and such efficiency enables accurate DFT calculations on systems of thousands and tens of thousands of atoms 25,26].

Single-point energy calculations were performed using the PBE gradient corrected exchange-correlation functional 27], along with norm-conserving pseudopotentials to describe the interactions between electrons and nuclei. Dispersion interactions were accounted for using a DFT+D approach 28,29]. Nonorthogonal generalised Wannier functional (NGWF) radii of 8 bohr were used for all atoms, and no truncation was applied to the density kernel. A kinetic energy cut-off of 880 eV was employed for each system. The interaction enthalpy (E_i_) of apoC-II(60-70) peptide on cyc(60-70), was determined as:



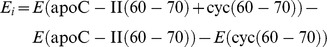
,

where *E*(apoC-II(60-70)+cyc(60–70)) is the total energy for the heterodimer complex, *E(*apoC-II(60–70)) is the total energy of apoC-II(60–70) and *E*(cyc(60–70)) is the total energy of the cyclic peptide in vacuum.

## Supporting Information

Figure S1 **Intra-molecular H-bond existence profile for cyc(60–70).** Red lines indicate H-bond existence between specific atoms which are tracked through the trajectory. The y-axis represents the total number of H-bonds formed. There are eight identifiable persistent H-bonds labelled with indices 83, 133, 154, 180, 233, 251, 252 and 276. These indices correspond to Gln70(NH)-Met60(O), Thr68(NH)-Thr62(O), Ile66(NH)-Thr64(O), Thr64(NH)-Ile66(O), Thr62(NH)-Thr68(O), Ser61(OG,HG)-Asp69(OD1), Ser61(OG,HG)-Asp69(OD2) and Met60(NH)-Gln70(O), respectively.
(TIF)

Figure S2 **Intra-molecular H-bond existence profile for apoC-II(60–70).** Red lines indicate H-bond existence between specific atoms which are tracked through the trajectory. The y-axis represents the total number of H-bonds formed. There are eight identifiable persistent H-bonds in the highlighted region with indices 108, 145, 147, 175, 190, 281, 305 and 306. These indices correspond to Asp69(NH)-Ser61(OG), Thr68(NH)-Met60(O), Thr68(NH)-Ser61(OG), Ile66(NH)-Thr62(OG1), Gly65(NH)-Thr62(O), Thr62(NH)-Ile66(O), Ser61(OG,HG)-Asp69(OD2) and Ser61(OG,HG)-Asp69(O), respectively. The highlighted simulated time period corresponds to β-hairpin like conformations that apoC-II(60–70) adopts (lin-c2). The substantial increase in H-bonds reveal that apoC-II(60–70) cannot adopt similar conformations like those observed in lin-c1 and lin-c3.
(TIF)

Figure S3 **Starting structures used in MD simulations.** Eight initial arrangements of the peptides, including its schematic and structural representations are shown. Arrangements 1 to 4 comprises the β-hairpin conformation identified from our previous study 10] and arrangements 5 to 8 comprises apoC-II(60–70) in its native conformation.
(TIF)
