# Supplementary figures and images for: "Janus" Cyclic Peptides: A New Approach to Amyloid Fibril Inhibition?
Source: PLoS One. 2013 Feb 20;8(2):e57437. doi: 10.1371/journal.pone.0057437 (PMC3577749; doi:10.1371/journal.pone.0057437)

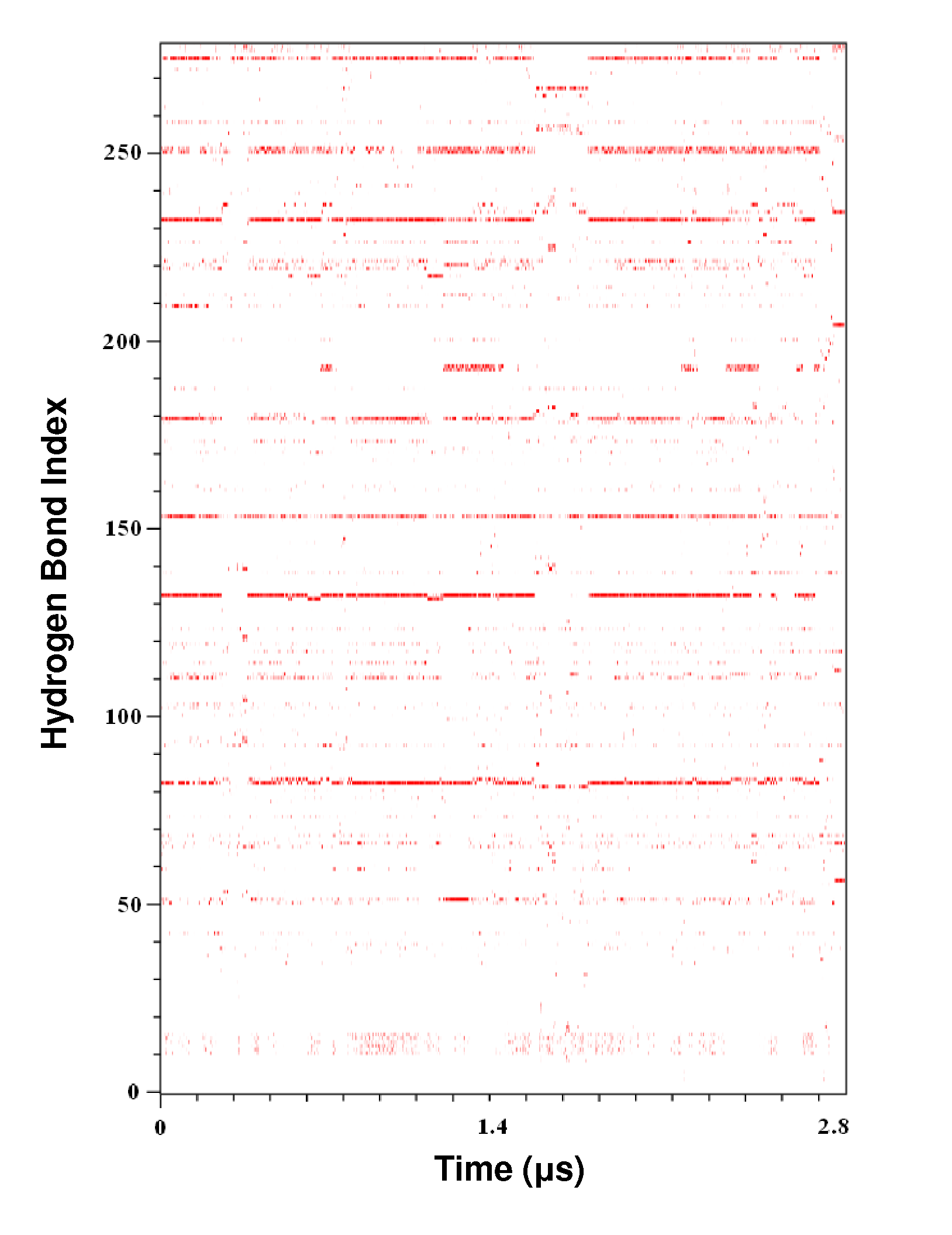

Supplement: Figure S1 — Intra-molecular H-bond existence profile for cyc(60–70). Red lines indicate H-bond existence between specific atoms which are tracked through the trajectory. The y-axis represents the total number of H-bonds formed. There are eight identifiable persistent H-bonds labelled with indices 83, 133, 154, 180, 233, 251, 252 and 276. These indices correspond to Gln70(NH)-Met60(O), Thr68(NH)-Thr62(O), Ile66(NH)-Thr64(O), Thr64(NH)-Ile66(O), Thr62(NH)-Thr68(O), Ser61(OG,HG)-Asp69(OD1), Ser61(OG,HG)-Asp69(OD2) and Met60(NH)-Gln70(O), respectively. (TIF) [file pone.0057437.s001.tif]

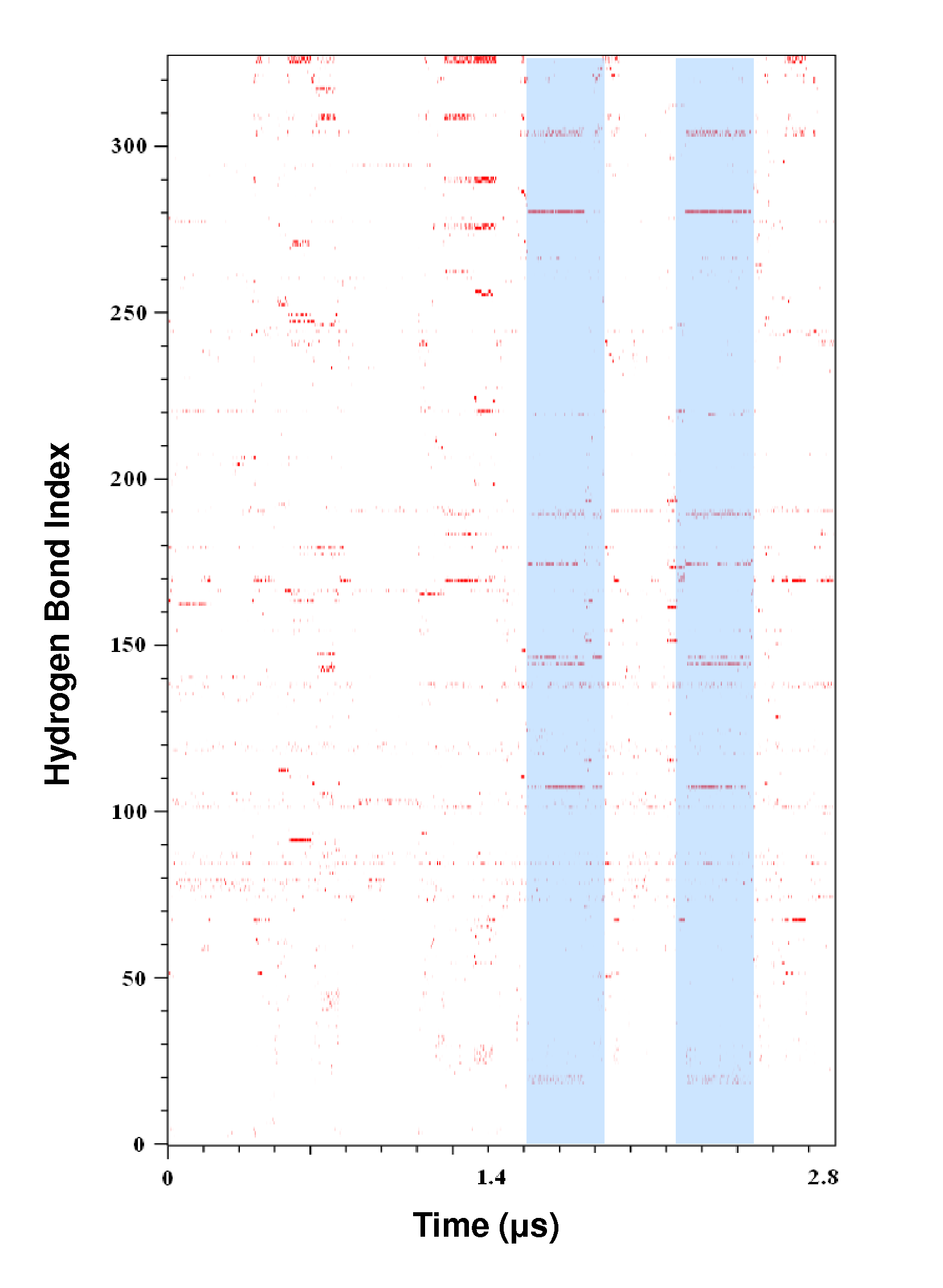

Supplement: Figure S2 — Intra-molecular H-bond existence profile for apoC-II(60–70). Red lines indicate H-bond existence between specific atoms which are tracked through the trajectory. The y-axis represents the total number of H-bonds formed. There are eight identifiable persistent H-bonds in the highlighted region with indices 108, 145, 147, 175, 190, 281, 305 and 306. These indices correspond to Asp69(NH)-Ser61(OG), Thr68(NH)-Met60(O), Thr68(NH)-Ser61(OG), Ile66(NH)-Thr62(OG1), Gly65(NH)-Thr62(O), Thr62(NH)-Ile66(O), Ser61(OG,HG)-Asp69(OD2) and Ser61(OG,HG)-Asp69(O), respectively. The highlighted simulated time period corresponds to β-hairpin like conformations that apoC-II(60–70) adopts (lin-c2). The substantial increase in H-bonds reveal that apoC-II(60–70) cannot adopt similar conformations like those observed in lin-c1 and lin-c3. (TIF) [file pone.0057437.s002.tif]

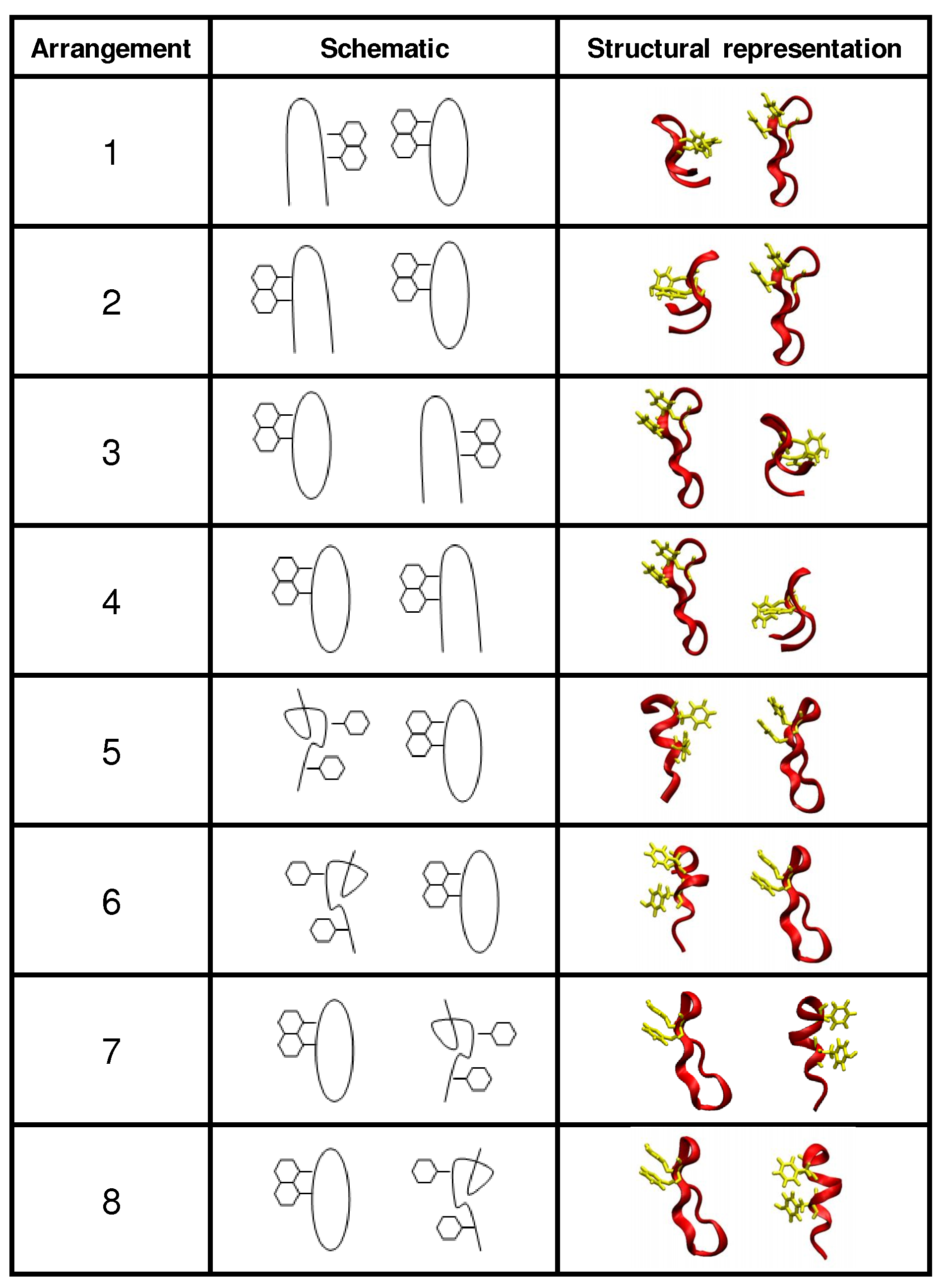

Supplement: Figure S3 — Starting structures used in MD simulations. Eight initial arrangements of the peptides, including its schematic and structural representations are shown. Arrangements 1 to 4 comprises the β-hairpin conformation identified from our previous study 10] and arrangements 5 to 8 comprises apoC-II(60–70) in its native conformation. (TIF) [file pone.0057437.s003.tif]
